# Tyrosine phosphorylation of Munc18c on residue 521 abrogates binding to Syntaxin 4

**DOI:** 10.1186/1471-2091-12-19

**Published:** 2011-05-06

**Authors:** Veronica Aran, Nia J Bryant, Gwyn W Gould

**Affiliations:** 1 Henry Wellcome Laboratory of Cell Biology, Davidson Building, Institute of Molecular, Cell and Systems Biology, College of Medical, Veterinary and Life Sciences, University of Glasgow. Glasgow G12 9QQ, UK; 2 The Physiological Laboratory, School of Biomedical Sciences, Crown Street, University of Liverpool, Liverpool L69 3BX, UK

## Abstract

**Background:**

Insulin stimulates exocytosis of GLUT4 from an intracellular store to the cell surface of fat and muscle cells. Fusion of GLUT4-containing vesicles with the plasma membrane requires the SNARE proteins Syntaxin 4, VAMP2 and the regulatory Sec1/Munc18 protein, Munc18c. Syntaxin 4 and Munc18c form a complex that is disrupted upon insulin treatment of adipocytes. Munc18c is tyrosine phosphorylated in response to insulin in these cells. Here, we directly test the hypothesis that tyrosine phosphorylation of Munc18c is responsible for the observed insulin-dependent abrogation of binding between Munc18c and Syntaxin 4.

**Results:**

We show that Munc18c is directly phosphorylated by recombinant insulin receptor tyrosine kinase *in vitro*. Using pull-down assays, we show that phosphorylation abrogates binding of Munc18c to both Syntaxin 4 and the v-SNARE VAMP2, as does the introduction of a phosphomimetic mutation into Munc18c (Y521E).

**Conclusion:**

Our data indicate that insulin-stimulated tyrosine phosphorylation of Munc18c impairs the ability of Munc18c to bind its cognate SNARE proteins, and may therefore represent a regulatory step in GLUT4 traffic.

## Background

Insulin stimulates glucose transport into fat and muscle by regulating translocation of the facilitative glucose transporter GLUT4 from an intracellular store to the plasma membrane (PM) [1]. In the absence of insulin, ~95% of cellular GLUT4 is sequestered within intracellular compartment(s), including specialised GLUT4 storage vesicles (GSVs)[1]. Upon insulin stimulation, GSVs traffic to the PM, resulting in a 10- to 20-fold increase in PM GLUT4 levels [1]. This is achieved by an increase in the rate of exocytosis and an inhibition of endocytosis [2].

GLUT4 is transported between various intracellular membrane-bound compartments by vesicular transport. Membrane traffic in all eukaryotic cells is controlled by specific SNARE complexes [3]. Members of the t-(target) family of SNARE proteins mark specific organelles. The formation of complexes between t-SNAREs and their cognate v-(vesicle) SNARE localised to the appropriate donor membrane is sufficient to catalyse bilayer fusion [4], thus SNAREs have been proposed to impart specificity to membrane traffic [3]. The t-SNARE complex consisting of Syntaxin4 and SNAP-23 (Sx4/S-23) controls fusion of GLUT4-containing vesicles with the PM, resulting in the final delivery of the transporter to the PM in response to insulin [1]. The predominant v-SNARE involved in this step is VAMP2 [1].

All SNARE complexes are subject to strict regulation to ensure that the trafficking steps they catalyse take place within the correct spatial and temporal coordinates. The Sec1/Munc18 (SM) family of proteins are key regulators of syntaxin function [5]. The SM protein that binds to Sx4 is Munc18c [1]. Adipocytes derived from MEFs from Munc18c knockout mice show enhanced GLUT4 translocation, suggesting that Munc18c inhibits insulin-stimulated externalization of GLUT4. This suggests that disruption of interaction between Sx4 and Munc18c might result in enhancement of insulin-stimulated GLUT4 translocation [6]. Consistent with this, over-expression of Munc18c was found to inhibit insulin-stimulated GLUT4 translocation in 3T3-L1 adipocytes, as did peptides designed to inhibit the binding of Munc18c to Sx4 [7,8]. Interestingly, Munc18c was found to inhibit the fusion of artificial liposomes mediated by Sx4/S-23 and VAMP2 *in vitro *[9], further supporting the notion of Munc18c inhibiting fusion of GLUT4 vesicles with the PM.

How may negative regulation of Sx4 by Munc18c be alleviated upon insulin treatment? Like many syntaxins, Sx4 adopts two distinct conformations, a closed conformer with the H_abc _domain folded back onto the SNARE domain (thus rendering this molecule unable to form functional SNARE complexes) and an open conformation with the H_abc _domain moved away from the SNARE domain. SM proteins have been shown to bind both of these conformations [10]: in mode 1, the central cavity of arch-shaped SM proteins cradle their cognate syntaxin in a closed conformation; in mode 2 the open conformation of syntaxin generates a free N-terminus which inserts into a hydrophobic pocket on the opposite face of the SM protein. Furthermore, it is now established that SM proteins can bind to their cognate SNARE ternary complex via a distinct mode (mode 3 binding), and it has been suggested that this mode of interaction between SM proteins and the SNARE machinery is of considerable importance [11,12]. Using an *in vitro *assay of fusion, the interaction of the SM protein Munc18a with its cognate SNARE complex accelerates fusion, with the SM protein making contact with both the t- and v-SNAREs [13]. By contrast, in the same kind of assay, Munc18c appears to act as an inhibitor of fusion [9]. Such data suggest the possibility that insulin may modulate one these binding modes between Sx4 and Munc18c to modulate fusion, but how this may be achieved is unknown.

Munc18c is known to become tyrosine phosphorylated (on Tyr-521) upon insulin stimulation of 3T3-L1 adipocytes [14], but whether Munc18c is a direct target of the insulin receptor tyrosine kinase or whether another kinase(s) is(are) responsible is unknown. Studies in β-cell lines have suggested phosphorylation of Munc18c may be a crucial trigger for insulin granule fusion with the PM [15]. Stimulation of 3T3-L1 adipocytes with insulin or platelet-derived growth factor (PDGF) promotes tyrosine phosphorylation of Munc18c, a modification that occurs concomitant with an observed dissociation of the Sx-4/Munc18c complex [16]. Expression of a mutant form of Munc18c (Y521A) was found to inhibit PDGF-stimulated GLUT4 translocation, suggesting that the tyrosine phosphorylation of Munc18c may impact the trafficking machinery involved in GLUT4 mobilisation. However, the effect of the Y521A mutant on insulin-stimulated GLUT4 trafficking remains unclear, since over-expression of both wild-type or mutant forms of Munc18c both inhibited GLUT4 translocation [16]. Although this latter study reveals that the Sx4/Munc18c complex is dissociated concomitant with tyrosine phosphorylation of Munc18c, whether this is a direct effect of tyrosine phosphorylation of Munc18c was not addressed, and little is known regarding the consequences of tyrosine phosphorylation on the ability of Munc18c to bind Sx4. To address this, we have used an *in vitro *approach to study the consequence of Munc18c phosphorylation on its binding to Sx4. We show that Munc18c is directly phosphorylated *in vitro *by recombinant insulin receptor tyrosine kinase, and that this phosphorylation occurs only on residue 521. Once phosphorylated, Munc18c is no longer able to bind monomeric Sx4 by either mode 1 or mode 2. Similarly, a phospho-mimetic mutant of Munc18c, Y521E, is unable to bind to either Sx4 or VAMP2. These data suggest that the tyrosine phosphorylation of Munc18c in response to insulin may trigger dissociation of Munc18c from Sx4, thus facilitating the formation of a SNARE complex for vesicle fusion.

## Results and Discussion

### Munc18c is directly phosphorylated by the insulin receptor in vitro

**Figure 1:**
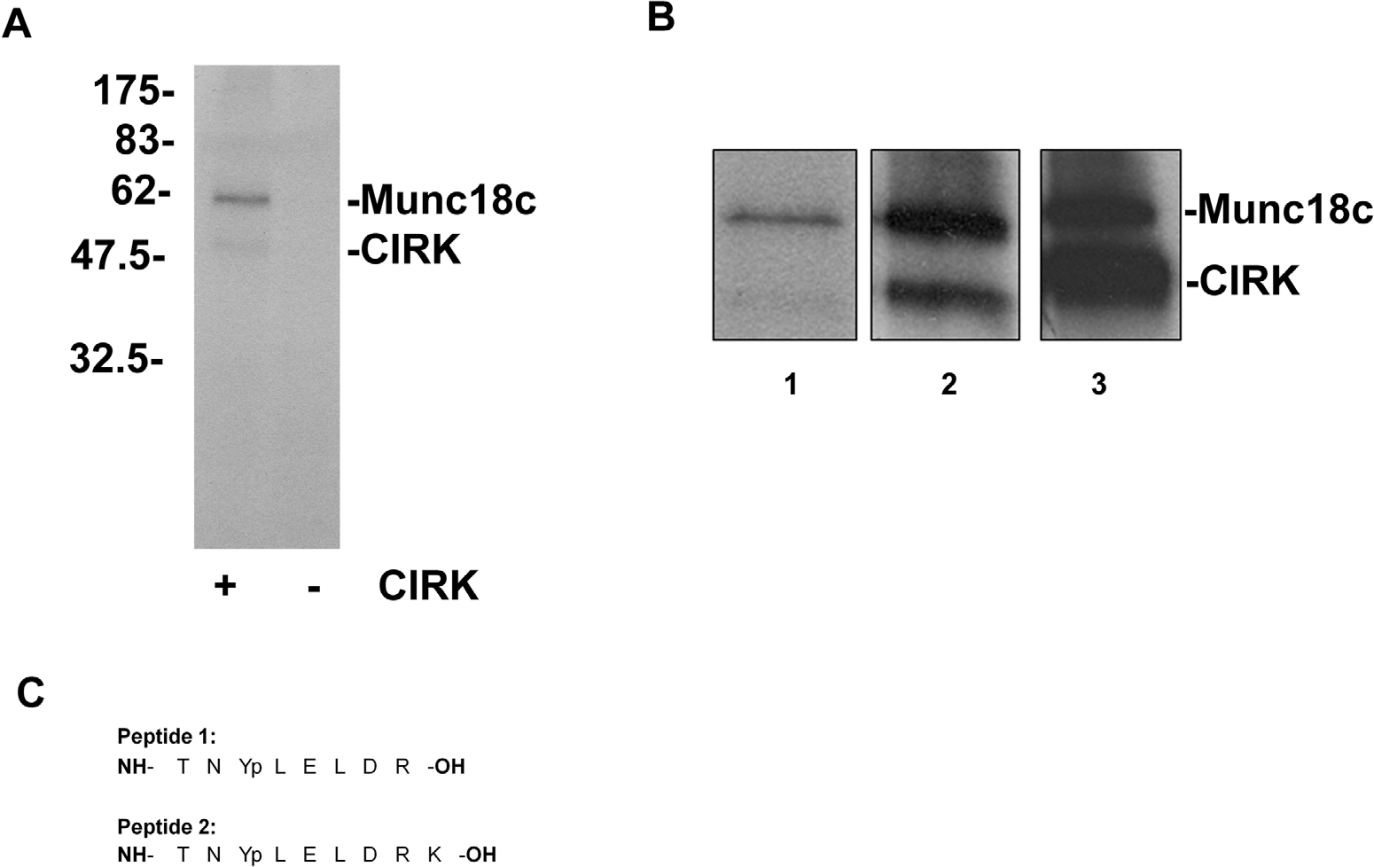
**Munc18c is a substrate for CIRK *in vitro***. ***A ***1 μg Munc18c was incubated with or without 370 ng CIRK in the presence of [γ-32P]-ATP. Reactions were stopped by boiling in Laemmli buffer prior to SDS-PAGE/autoradiography. Shown are data from a representative experiment. ***B ***1 μg Munc18c was incubated with 370 ng CIRK in the presence of [γ-^32^P]-ATP for 30 minutes (lane 1), 120 minutes (lane 2), or 150 minutes with 1.8 μg CIRK (lane 3). Reactions were stopped as in ***A***. Note that different exposures of the autoradiograms are shown for clarity. ***C ***Two phosphopeptides were identified by Mass Spec analysis of phosphorylated Munc18c (peptide 2 represents mis-cleavage of peptide 1).

A proteomics based screen of insulin-stimulated tyrosine phosphorylated proteins in 3T3-L1 adipocytes revealed that Munc18c exhibits a >10-fold increase in tyrosine phosphorylation on residue 521 [14]. As this screen did not inform on the extent of tyrosine phosphorylation of any identified target nor reveal whether the kinase response was the insulin receptor itself, we sought to determine whether the insulin receptor can directly phosphorylate Munc18c and if so, to what extent. To address this, we employed a recombinant protein corresponding to residues 941-1343 of the β-subunit of the insulin receptor, the region containing the tyrosine kinase domain (referred to as Cytosolic Insulin Receptor Kinase, CIRK) [17]. This protein, when expressed and purified from baculovirus-infected Sf9 cells, mimics at least some of the reactions catalysed by the insulin receptor itself [17,18]. Using an *in vitro *kinase assay, we found that CIRK phosphorylates recombinant Munc18c in a time- and dose-dependent manner, such that by 150 minutes ~30% of the Munc18c in the reaction was phosphorylated (Figure 1). We next determined which residue(s) of Munc18c are phosphorylated by CIRK *in vitro *by subjecting the samples to mass spectrometry (Additional file 1). This revealed that the only residue of Munc18c phosphorylated by CIRK was Tyrosine-521 (Figure 1), unequivocally demonstrating that, at least *in vitro*, Munc18c is a direct target of the insulin receptor.

### Phosphorylation of Munc18c abrogates binding to syntaxin 4 and VAMP2

**Figure 2:**
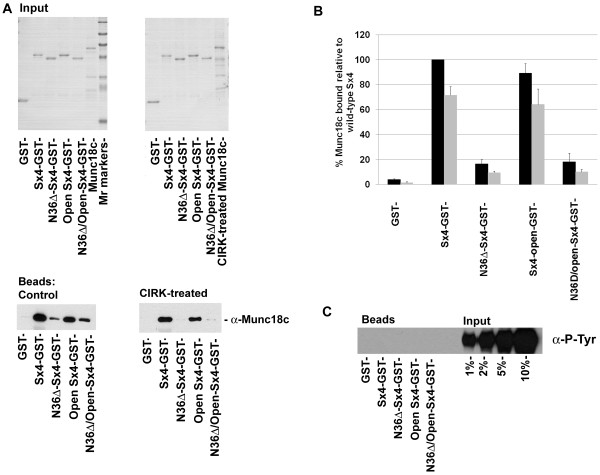
**Munc18c phosphorylated by CIRK no longer binds Sx4**. ***A ***1 μg of GST, Sx4-GST or the Sx4 mutants immobilised on glutathione-Sepharose were incubated with 1 μg of Munc18c (previously treated with or without 370 ng CIRK for 150min) in binding buffer overnight at 4°C. 6% of the samples were removed and subjected to SDS-PAGE and Coomassie stained to assess inputs (upper panels). Sepharose beads were washed prior to immunoblot analysis with anti-Munc18c (10% of eluted material; lower panels). Data shown are from representative experiments, repeated three times with similar results. ***B ***Shows quantification of the data from 3 experiments of this type. Levels of control (non-phosphorylated) Munc18c bound to the different Sx4 species is expressed as a % of the level bound to wild-type Sx4 (black bars), (mean ± SD). Data from phosphorylated Munc18c (grey bars) is also shown. Note that the level of binding of phosphorylated Munc18c to wild-type Sx4 is decreased by ~30%. Quantification of the extent of phosphorylation of Munc18c under these conditions revealed that a roughly equivalent fraction of Munc18c is phosphorylated. See text for details. The amount of Munc18c captured by the different Sx4 species is in good agreement with published data [19]. ***C ***The material eluted from the beads was also probed with anti-phosphotyrosine antibodies (40% of eluted material loaded per lane) as labelled. In this case, the indicated amounts of input were also loaded in order to confirm the presence or absence of any tyrosine phosphorylated Munc18c in the GST pull-down. Data shown are from representative experiments, repeated three times with similar results.

Munc18c binds both the open and closed conformations of Sx4, as well as to the v-SNARE, VAMP2 [19]. We therefore sought to determine whether phosphorylation of Munc18c modulates these interactions. Four different GST-tagged Sx4 constructs immobilised on glutathione-Sepharose were employed [19]: wild-type Sx4, Sx4-open (a L173A/E174A mutant of Sx4 which adopts an open conformation), Sx4 lacking the N-terminal 36 amino acids (required for mode 2 binding to Munc18c; N36Δ) and a double mutant (open plus N36Δ). These were incubated with Munc18c pre-incubated with or without CIRK (Figure 2A). After incubation, the amount of Munc18c associated with the Sx4-proteins was determined by immunoblotting. Figure 2A shows that Munc18c was readily detected in complex with the wild-type GST-Sx4 and the 'open' mutant, but was dramatically reduced for the N36Δ and double mutants, in agreement with previous studies [19]. Following pre-incubation with CIRK, a reduction in the amount of Munc18c in complex with the Sx4 species was evident (Figure 2A, quantified in Figure 2B).

In order to ascertain whether this reduction is a consequence of tyrosine phosphorylation of Munc18c, we performed immunoblot analysis using anti-phosphotyrosine antibodies (Figure 2C). This revealed that none of the tyrosine phosphorylated Munc18c was in association with any of the GST-Sx4 species examined. Under the conditions employed, CIRK treatment resulted in tyrosine phosphorylation of ~30% of the input Munc18c. Given that tyrosine phosphorylated Munc18c cannot bind Sx4 (Figure 2C) this explains why the levels of Munc18c in association with Sx4, detected by anti-Munc18c, are reduced upon CIRK treatment by ~30%, a figure in close agreement with the extent of Munc18c phosphorylation observed under these conditions (Figure 2A, quantified in Figure 2B).

**Figure 3:**
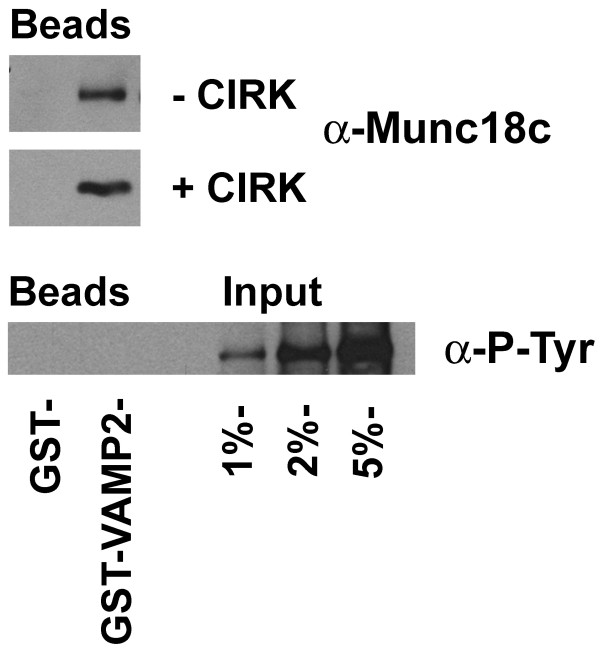
**Munc18c phosphorylated by CIRK no longer binds VAMP2**. 1 μg of GST or GST-VAMP2 immobilised on glutathione-Sepharose were incubated with 1 μg of Munc18c (which had been previously treated with or without 1.8 μg CIRK for 150min) in binding buffer overnight at 4°C. Sepharose beads were washed prior to immunoblot analysis with anti-Munc18c (upper panel) or anti-phosphotyrosine antibodies (lower panel) as labelled. In the case of the anti-phosphotyrosine immunoblot, the indicated amounts of input were also loaded to reveal the presence or absence of any tyrosine phosphorylated Munc18c in the GST pull-down. Data shown are from representative experiments, repeated three times with similar results.

We have previously reported that Munc18c binds the v-SNARE VAMP2 [19]. We therefore examined whether this interaction is modulated by Munc18c tyrosine phosphorylation (Figure 3). Although Munc18c readily bound GST-VAMP2, none of the pulled-down protein was immuno-reactive with anti-phosphotyrosine antibodies. Collectively, our data suggest that Munc18c is unable to interact with either its cognate syntaxin (Sx4) or VAMP2 when phosphorylated on Y521.

### A phosphomimetic mutant of Munc18c is unable to bind Syntaxin 4 or VAMP2

**Figure 4:**
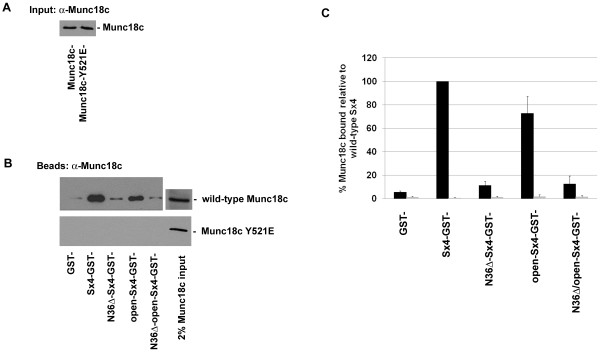
**Munc18c-Y521E does not bind to Sx4**. 1 μg of GST, Sx4-GST or the Sx4 mutants immobilised on glutathione-Sepharose, were incubated with 1 μg of either wild-type Munc18c or Munc18c-Y521E in binding buffer overnight at 4°C. An example of the input Sx4 species is shown in Figure 2. ***A ***shows an anti-Munc18c immunoblot of wild-type Munc18c and Munc18c-Y521E recombinant proteins (3% of input in each case), indicating that both species are equally recognised by the anti-Munc18c antibody used. Sepharose beads were washed prior to immunoblot analysis with anti-Munc18c (panel ***B***; 2% of input Munc18c or Munc18c-Y521E is also shown). Data from a representative experiment is shown, repeated three times with quantitatively similar results. We also repeated these experiments using increasing amounts of Munc18c (or the Y521E mutant) between 1 and 10 μg incubated with either 0.2 or 1.0 μg of Sx4-GST. We saw no binding of Munc18c-Y521E at any of these conditions (data not shown). Quantification of these experiments is shown graphically in panel ***C***, black bars are wild-type Munc18c binding, grey bars are Munc18c-Y521E binding. Data presented as a % of wild-type Munc18c binding wild-type Sx4 (mean ± SD).

To substantiate these findings, we made a mutant of Munc18c with Y521 replaced by the (relatively) bulky negative residue glutamate (Y521E); although not aromatic, the side chain of glutamate is the biggest of the negatively charged amino acids and thus we hoped would function as a phosphomimetic. The characteristics of this protein were examined. The mutant was recognised by anti-Munc18c and exhibited the expected molecular weight (Figure 4A). The wild-type and mutant proteins exhibited similar far UV circular dichroism spectra, suggesting that Munc18c-Y521E exhibited little change in overall secondary structure compared to the wild-type protein, a conclusion further supported by the similar intrinsic tryptophan fluorescence spectra of the two proteins (data not shown). We therefore repeated the binding studies of Figure 2, this time using Munc18c-Y521E or wild-type Munc18c. As shown in Figure 4, we were unable to detect any binding of Munc18c-Y521E to Sx4 (or VAMP2, data not shown). In experiments not shown, we were unable to detect any interaction between Munc18c-Y521E and Sx4 even with a ~50-fold excess of Munc18c over Sx4 (see legend to Figure 4), or when recombinant Munc18c-Y521E was immobilised to Ni-Agarose and incubated with recombinant Sx4.

These data support the notion that phosphorylation of Munc18c on Y521 may represent an insulin-controlled regulatory mechanism. Although the structure of Munc18c has been largely solved [20], Y521 is in an unstructured area, probably a flexible domain, located near the surface of the molecule. The structure reveals that Y521 is close to the hydrophobic pocket into which the N-terminus of Sx4 inserts in mode 2 binding [20]. However, this would not explain why CIRK-phosphorylated Munc18c or Munc18c-Y521E do not bind either closed Sx4 or VAMP2. It is possible that the phosphorylation of Y521 may cause a more significant structure change in Munc18c, abrogating all binding modes, but CD and fluorescence analysis of the mutant suggests this is unlikely. Regardless, our data suggest that Y521 phosphorylation of Munc18c abrogates binding to monomeric Sx4 and VAMP2, and perhaps plays a role in insulin-stimulated SNARE-mediated delivery of GLUT4 to the PM. Studies in which this mutant of Munc18c is expressed in adipocytes at levels similar to wild-type protein in the absence of the endogenous Munc18c will be important. At present, such studies are proving technically challenging, as over-expression of even wild-type Munc18c significantly impairs GLUT4 translocation [8]. Given our incomplete understanding of the functional role played by the different modes of Munc18 binding, a future assessment of the consequences of Munc18c phosphorylation on mode 3 binding to its cognate SNARE ternary complex will be informative.

## Conclusions

The data presented support the hypothesis that Y521 phosphorylation of Munc18c by the insulin receptor tyrosine kinase abrogates binding to monomeric Sx4 and VAMP2, suggesting that this event may play a role in insulin-stimulated SNARE-mediated delivery of GLUT4 to the PM.

## Methods

### Recombinant protein expression

pQE30-Munc18c was used as template for the Y521E-coding mutant which was fully sequenced on both strands. His-6-Munc18c and His-6-Munc18c-Y521E were expressed in *E. coli *M15 cells and purified using Ni-NTA-agarose as described [9]. The recombinant Sx4 species and GST-VAMP2 were expressed and purified as reported [9,19]. Note that the Sx4-GST fusions employed in this study encompass the entire cytosolic domain of the Sx4 (residues 1-273) with the GST added at the C-terminus of the Sx4. As we have previously reported, in this construct, the GST-moiety does not interfere with binding of Munc18c [19]. The Sx4-open mutant and the other mutants were those characterised in [19].

### Pull-down assays

1 μg GST-Sx4 (or mutants thereof), immobilised on glutathione-Sepharose, was incubated overnight with 1 μg recombinant Munc18c (pre-treated with or without cytosolic insulin receptor kinase) or 1 μg recombinant Munc18c-Y521E in 300 μl of binding buffer (20 mM Hepes pH 7.4, 150 mM potassium acetate, 1 mM MgCl_2 _and 0.05% (v/v) Tween-20) at 4°C with rotation [19]. After removal of a sample to assess input levels, unbound protein was removed by three washes in binding buffer, followed by three washes in binding buffer containing 0.2% (w/v) fish tail gelatin, three washes in binding buffer containing 5% (w/v) glycerol and three washes in binding buffer alone. Beads were resuspended in Laemmli sample buffer, boiled for 5 minutes and bound proteins by SDS-PAGE and/or immunoblotting.

### CIRK phosphorylation assays

Recombinant CIRK [17] was provided by Professor Gustav E. Lienhard (Dartmouth Medical School). 1 ml recombinant CIRK (8 mg/ml) was activated by incubation with an equal volume of 100 mM Hepes pH 7.5, 4 mM sodium ATP, 5 mM MnCl_2 _and incubated at 30°C for 30 minutes. CIRK was then diluted 10-fold into 50 mM Hepes pH 7.5 containing 10% (v/v) glycerol and 1 mM dithiotheritol [17]. At this stage, CIRK was at 0.4 mg/ml and the specific activity was assayed to be 46 nmol/min/mg. Samples were stored at -80°C with no loss of activity over the periods of these experiments.

For *in vitro *kinase assays, CIRK was mixed with Munc18c (1 μg) in 50 μl kinase buffer (50 mM Hepes pH 7.5, 4 mM MnCl_2_, 0.2 mM DTT and 100 μM Na-ATP), and incubated at 30°C for the times indicated. In some experiments, this was supplemented with 6 μCi [γ-^32^P]-ATP to allow the stoichiometry of phosphorylation to be determined. Monoclonal anti-phosphotyrosine antibodies (PT-66) were from AbCam (Cambridge, UK).

### Mass spectrometry

Analysis of the sites of phosphorylation of Munc18c by CIRK was performed at the Dundee University Post-Genomics Facility. After incubation of 1 μg Munc18c with CIRK for 30 minutes at 30°C, the sample was trypsinised and the phosphorylated sites determined by liquid chromatography-electrospray ionisation-mass spectrometry (LC-ESI-MS) using an ABI QTrap 4000 instrument. This generated two major [M-2H]^2- ^(double charged) quase molecular ions at *m*/*z *551.3 and 615.3 which were absent from the control Munc18c sample that was not incubated with CIRK, suggesting that they represent phosphorylated tryptic peptides. After identification, the same ions were submitted to ESI-MS/MS to determine their amino acid sequence and to locate the phosphorylated residue.

## Abbreviations

CIRK: cytoplasmic insulin receptor kinase; GLUT: glucose transporter; MEF: mesenchymal embryonic fibroblasts; PM: plasma membrane; S-23: SNAP23; SM: Sec1/Munc18; SNARE: soluble NSF attachment protein receptor; Sx4: Syntaxin 4; GSV: Glut4-storage vesicle;

## Authors' contributions

VA performed the experiments and wrote the paper. NJB and GWG analysed the results and wrote the paper. All authors read and approved the final manuscript.

## Supplementary Material

Additional file 1 **Mass Spec analysis of phosphorylated Munc18c**. **Upper panel**: 1 μg of Munc18c was incubated with CIRK for 30 minutes as described in *materials and methods*. The reaction was stopped, the sample trypsinised and analysed by LC-ESI-MS in a Qtrap 400 in negative ion mode. The spectrum shows only the ions detected by the precursor ion scan of 79 mass units which represents PO_3_^- ^and suggest a phosphopeptide. The insert shows a detail of the two major [M-2H]^2- ^ions (peptide 1 and 2). The peptides are accompanied by the same peptide coupled to one or two metal ions (i.e. *m/z *562.3, 569.3 and 580.3 for peptide 1 and 625.3, 633.3 and 644.4 for peptide 2). **Lower panel**: Exactly as upper panel except Munc18c was incubated in CIRK buffer for 30 minutes in the absence of CIRK (i.e. non-phosphorylated control). Click here for file
