# Supplementary figures and images for: Tyrosine phosphorylation of Munc18c on residue 521 abrogates binding to Syntaxin 4
Source: BMC Biochem. 2011 May 6;12:19. doi: 10.1186/1471-2091-12-19 (PMC3103433; doi:10.1186/1471-2091-12-19)

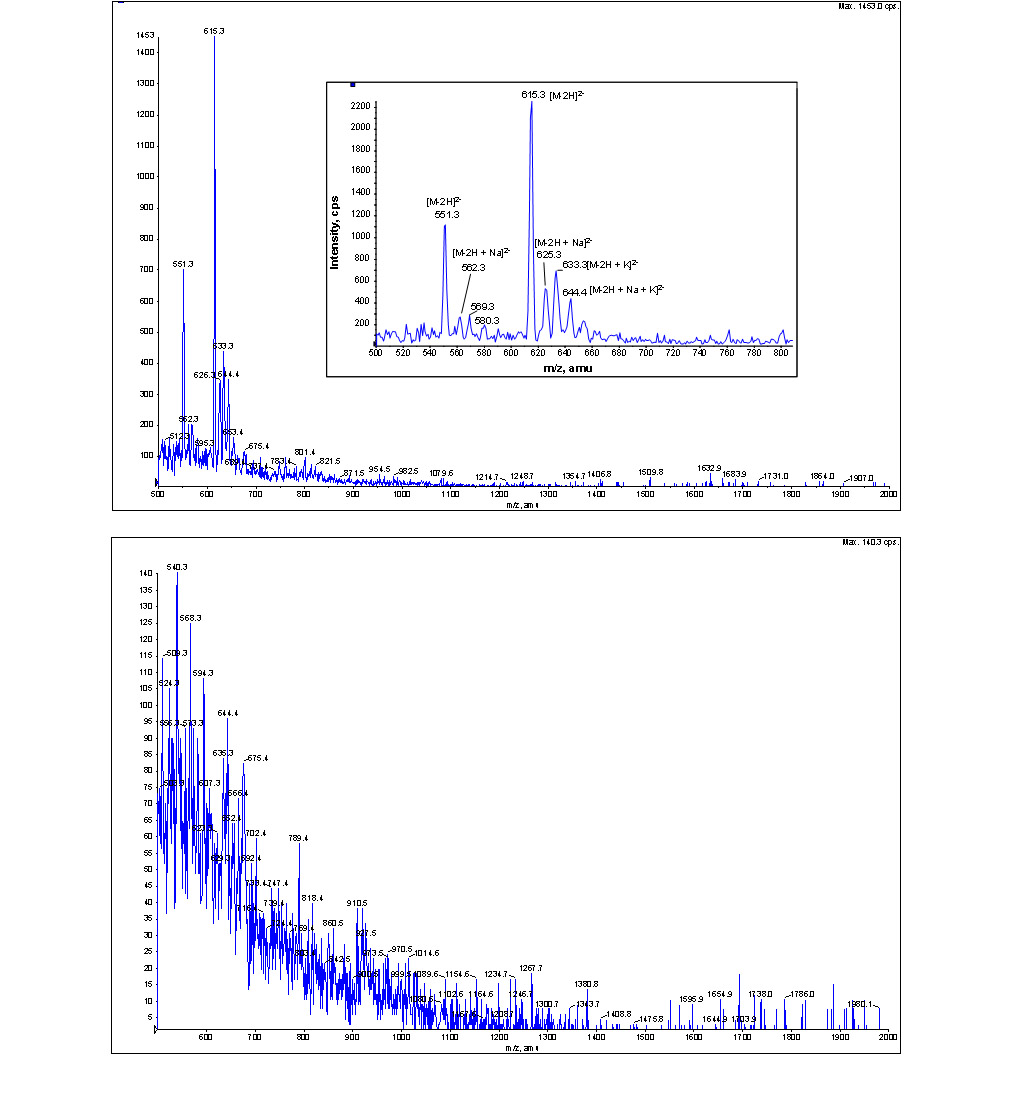

Supplement: Additional file 1 — Mass Spec analysis of phosphorylated Munc18c. Upper panel: 1 μg of Munc18c was incubated with CIRK for 30 minutes as described in materials and methods. The reaction was stopped, the sample trypsinised and analysed by LC-ESI-MS in a Qtrap 400 in negative ion mode. The spectrum shows only the ions detected by the precursor ion scan of 79 mass units which represents PO3- and suggest a phosphopeptide. The insert shows a detail of the two major [M-2H]2- ions (peptide 1 and 2). The peptides are accompanied by the same peptide coupled to one or two metal ions (i.e. m/z 562.3, 569.3 and 580.3 for peptide 1 and 625.3, 633.3 and 644.4 for peptide 2). Lower panel: Exactly as upper panel except Munc18c was incubated in CIRK buffer for 30 minutes in the absence of CIRK (i.e. non-phosphorylated control). [file 1471-2091-12-19-S1.JPEG]
